# Predictive factors for bacteremia in febrile infants with urinary tract infection

**DOI:** 10.1038/s41598-020-61421-4

**Published:** 2020-03-11

**Authors:** Seo Hee Yoon, HyunDo Shin, Keum Hwa Lee, Moon Kyu Kim, Dong Soo Kim, Jong Gyun Ahn, Jae Il Shin

**Affiliations:** 1 Department of Pediatrics, Yonsei University College of Medicine, Yonsei-ro 50, Seodaemun-gu, C.P.O. Box 8044, Seoul, 120-752 Korea; 2 Division of Pediatric Nephrology, Severance Children’s Hospital, Seoul, 03722 Korea; 3 Institute of Kidney Disease Research, Yonsei University College of Medicine, Seoul, 03722 Korea

**Keywords:** Predictive markers, Urinary tract infection, Risk factors, Fever

## Abstract

This study aimed to investigate the predictive factors of concomitant bacteremia occurring in febrile infants who initially presented with pyuria and fever, and were subsequently diagnosed with culture-proven urinary tract infection (UTI). We conducted a retrospective cohort study for January 2010–October 2018 that included infants younger than six months with febrile UTI at a tertiary hospital. The study included 463 patients, of whom 34 had a concomitant bacteremic UTI. Compared to those in the non-bacteremic urinary tract infection (UTI) group, the bacteremic UTI group had a lower mean age; higher levels of C-reactive protein (CRP), delta neutrophil index (DNI, reflects the fraction of immature granulocytes) and blood urea nitrogen (BUN); lower levels of hemoglobin (Hb) and albumin; and a lower platelet count. Vesicoureteral reflux (VUR) was detected nearly twice as often in patients with bacteremic UTI compared to those with non-bacteremic UTI (59.3% vs. 30.6%; *P* = 0.003). Univariate logistic analyses showed that age ≤90 days; higher DNI, CRP, and creatinine levels; lower Hb and albumin levels; and the presence of VUR were predictors for bacteremic UTI. On multivariate logistic regression analysis, age ≤90 days, higher DNI and CRP levels, and the presence of VUR were independent predictors of bacteremic UTI. The area under the receiver operating characteristic curve of the multivariate model was 0.859 (95% CI, 0.779–0.939; *P* < 0.001). Age ≤90 days, higher DNI and CRP values may help predict bacteremia of febrile infants younger than 6 months with UTI. Vesicoureteral reflux imaging is also recommended in infants with bacteremic UTI to evaluate VUR.

## Introduction

Urinary tract infection (UTI) has been described as the most common serious bacterial infection in febrile infants^1^. The rate of concomitant bacteremia in febrile UTI varies from 5% to 31%, depending on various locations and patient cohorts^2,3^. Although a recent study has shown that outcomes were good among infants with bacteremic UTI^4^, infants with concomitant bacteremic UTI are still prone to adverse outcomes such as prolonged admission, shock, bacterial meningitis, and intensive-care unit admission^5^. Early diagnosis and prompt decision-making on the initial treatment of young febrile infants suspected of having bacteremia are important but not feasible because confirming positive blood cultures is usually time-consuming.

Several factors have been previously reported to predict bacteremic UTI in pediatric patients: younger age^3,6^; clinical presentation such as ill appearance^5,7^ and feeding problems^8^; laboratory parameters including increased creatinine levels^9,10^; high levels of inflammatory markers such as white blood cell (WBC) count^5^, presence of bands^5^ and C-reactive protein (CRP)^7,10,11^; and genitourinary (GU) tract malformations^8^.

However, the clinical signs or symptoms of UTI are usually vague and unspecific in young infants^12^. Clinically determining whether children have serious complications when they may not appear ill is difficult for physicians^13^. Pantell *et al*. reported that among 3,066 febrile infants aged ≤3 months, only 58.1% of the infants with bacteremia or bacterial meningitis appeared ill^14^. In addition, Roman *et al*. reported no major clinical differences between infants with bacteremic and non-bacteremic UTI at presentation^4^. Therefore, objective predictors will be more useful to identify concomitant bacteremia at the initial presentation. Still, the predictive value of inflammatory markers such as WBC, total band count, CRP, and serum creatinine level remains controversial because of discordant results^4,6,9,15,16^. Furthermore, GU abnormality showed no statistical significance in terms of predicting concomitant bacteremia/sepsis in recent studies^9,10^.

Preceding pediatric studies have reported that the prevalence of concomitant bacteremic UTI was mostly limited to infants younger than six months^4,6^. The prevalence of UTI in infants aged 3–6 months was similar to that in those younger than three months in a previous meta-analysis^17^; nonetheless, studies that assessed the risk factors in predicting bacteremic UTI were mainly focused on infants younger than three months^5,7,10,11^.

This study aimed to investigate the initial risk factors for bacteremia in infants aged younger than six months with febrile UTI at presentation, confirming previously established parameters and identifying other new laboratory findings.

## Methods

This retrospective cohort study was performed in one tertiary referral hospital from January 2010 to October 2018. The researchers reviewed the medical data of infants ≤ 6 months initially presenting with fever (≥38.0 °C) and pyuria and eventually diagnosed as having culture-proven UTI after admission. Pyuria was defined by urine WBC count >5 cells/high power field (HPF)^18^. UTI was defined on the basis of the urine culture results: from a catheter urine specimen culture with a colony count ≥50,000 or from a bag-collected specimen culture with ≥100,000 colonies^4,18^. Cases were excluded when two or more organisms were isolated on culture.

Data on patient age and sex, route of admission, duration of fever and hospitalization, bacterium isolated from urine and blood cultures, initial laboratory findings, the presence of vesicoureteral reflux (VUR; confirmed by voiding cystourethrography [VCUG]/radionuclide cystoscopy/video urodynamic studies^19^), and ^99m^technetium dimercaptosuccinic acid (DMSA) renal scan results during the acute stage (within seven days from admission) were collected from the medical records. DMSA renal scan findings were classified according to the previous study^20^.

At the participating hospital, blood and urine cultures are routinely performed for febrile patients admitted with UTI. At least two sets of blood cultures were drawn before antibiotic therapy. Concomitant bacteremic UTI is defined when the same bacterium is isolated from both urine and blood cultures in the same patient within 48 h^9^. Only patients for whom both urine culture and blood culture were performed were included in this study.

Follow-up data including urinalysis at six months and one year after admission, and DMSA renal scan six months after admission^21^ were obtained. Estimated glomerular filtration rate (eGFR) is currently the most common way of assessing the degree of kidney impairment and following the course of a disease^22^. However, eGFR is not routinely examined for febrile patients admitted with UTI at our institution. Therefore, we have retrospectively reviewed the medical records of enrolled patients; if the results of eGFR were present after index UTI, we obtained the last result for eGFR and its date of testing to calculate the follow-up time from the index UTI admission. We calculated the cystatin C-based eGFR using the Chronic Kidney Disease in Children study (CKiD)-derived equation^23^. An eGFR ≥ 90 mL/min/1.73 m^2^ was regarded as normal^24,25^.

### Statistical analyses

Patients with bacteremic UTI were compared to those with non-bacteremic UTI. Demographics, clinical, and laboratory data were presented as mean ± standard deviation or frequency. Categorical variables were compared using the chi-squared test. Continuous variables were compared using the Student t-test or Mann–Whitney test, as appropriate. Univariate and multivariate binary logistic regression analyses were performed to identify the significant risk factors for bacteremic UTI, and the odds ratio (OR) and 95% confidence interval (CI) were calculated. If variables were found to be significant in the univariate analyses (*P* < 0.05), they were included in the multivariate regression analysis.

Receiver operating characteristic (ROC) curves were analyzed by estimating the area under the curve (AUC) to examine the predicting capacity of laboratory parameters and multivariate model in bacteremic UTI. The optimal cutoff values were calculated on the basis of the Youden index. Statistical analyses were performed using SPSS version 23.0 for Windows (SPSS Inc., Chicago, IL, USA) and MedCalc Statistical Software version 18.6 (MedCalc Software, Ostend, Belgium). *P* < 0.05 was considered significant.

### Ethical approval

This study was approved by the Institutional Review Board at Yonsei University Health System (approval number: 4-2018-1086). All research was performed in accordance with relevant guidelines and regulations. The requirement for written informed consents was waived by the Institutional Review Board due to retrospective study design.

## Results

### Baseline characteristics

During the study period, 463 cases of UTI that fully met inclusion criteria were enrolled. Among them, 34 were determined to be bacteremic UTI. The mean age was 3.6 ± 1.4 months, and 344 (74.3%) were boys, and 182 (39.3%) were younger than 90 days (Table 1).

**Table 1:** Comparison between infants with bacteremia and non-bacteremia with febrile urinary tract infection at admission.

Variables	Bacteremic UTI (n = 34)	Non-bacteremic UTI (n = 429)	*P*-value
Admission route			0.37^a^
ED	29 (85.3%)	338 (78.8%)	
OPD	5 (14.7%)	91 (21.2%)	
Urine samples			0.745^a^
Catheterization	4 (11.8)	59 (13.8)	
Urine bag	30 (88.2)	370 (86.2)	
Age (month)	2.8 ± 1.5	3.6 ± 1.4	<0.001^b^
Age ≤ 90 days	24 (70.6%)	158 (36.8%)	<0.001^a^
Male sex (%)	22 (64.7%)	322 (75.1%)	0.184^a^
Length of stay (days)	7.4 ± 3.9	4.8 ± 1.7	0.001^c^
Duration of fever (days)			
Total duration	2.8 ± 1.5	2.6 ± 1.6	0.573^b^
Before admission	1.6 ± 0.9	2.1 ± 1.3	0.032^b^
WBC (/μL)	13.9 ± 7.7	15.4 ± 5.4	0.268^c^
ANC (/μL)	8.3 ± 5.3	8.4 ± 4	0.840^b^
Hb (g/dL)	10.5 ± 0.8	10.9 ± 1	0.021^b^
Platelet count (k/μL)	411 ± 151	458 ± 127.7	0.043^b^
CRP (mg/L)	66.3 ± 42.9	42.4 ± 33.7	<0.001^b^
DNI (%)	4.1 ± 6.3	0.9 ± 2.1	0.006^c^
ESR (mm/H)	39.6 ± 26.5	36.6 ± 26.8	0.549^b^
Na (mmol/L)	136.7 ± 4.2	137.4 ± 2.2	0.084^b^
AST (IU/L)	31.6 ± 14.6	36.3 ± 35.4	0.444^b^
ALT (IU/L)	27.5 ± 21.6	29.4 ± 34.2	0.756^b^
Albumin (g/dL)	3.8 ± 0.3	3.9 ± 0.3	0.002^b^
Total bilirubin (mg/dL)	0.8 ± 0.7	0.6 ± 0.7	0.169^b^
BUN (mg/dL)	11.4 ± 5.7	8.2 ± 4.8	<0.001^b^
Cr (mg/dL)	0.3 ± 0.2	0.2 ± 0.1	0.125^c^
Urine nitrite test (+)	22 (64.7%)	226 (52.7%)	0.176^a^

Data are presented as case numbers, percentages, or the mean ± standard deviation. Statistically significant differences were demonstrated using ^a^Chi-square test, ^b^Student t-test, and ^c^Mann–Whitney test. ANC, absolute neutrophil count; AST, aspartate aminotransferase; ALT, alanine aminotransferase; BUN, blood urea nitrogen; Cr, Creatinine; CRP, C-reactive protein; ED, emergency department; ESR, erythrocyte sedimentation rate; Hb, hemoglobin; Na, sodium; OPD, outpatient department; UTI, urinary tract infection; WBC, white blood cell.

### Clinical and laboratory differences between bacteremic and non-bacteremic UTI

The mean age of the bacteremic UTI group (2.8 ± 1.5 months; range, 1.2–5.7 months) was younger than that of the non-bacteremic UTI group (3.6 ± 1.4 months; range, 1.0–6.0 months) (*P* < 0.001). The bacteremic UTI group exhibited shorter fever duration before admission (*P* = 0.032); higher CRP level (*P* < 0.001), delta neutrophil index (DNI), which reflects the level of immature granulocytes (*P* = 0.006), and blood urea nitrogen (BUN) (*P* < 0.001); and lower levels of hemoglobin (Hb)(*P* = 0.021), albumin (*P* = 0.002), and platelet counts (*P* = 0.043); No significant differences were found in sex, total duration of fever, WBC, absolute neutrophil count, erythrocyte sedimentation rate, sodium, creatinine, aspartate aminotransferase (AST), alanine aminotransferase (ALT), total bilirubin, or presence of a positive urine nitrite test (Table 1). *Escherichia coli* was the most commonly isolated organism in both bacteremic and non-bacteremic UTI (82.4% and 87.6%, respectively) (Table 2).

**Table 2:** Bacterial strains isolated from blood and urine cultures.

Bacterial isolates	Bacteremic UTI (n = 34)	Non-bacteremic UTI (n = 429)
*Citrobacter freundii*	0 (0.0%)	4 (0.9%)
*Citrobacter koseri*	0 (0.0%)	1 (0.2%)
*Enterobacter aerogenes*	3 (8.8%)	14 (3.3%)
*Enterobacter cloacae*	2 (5.9%)	4 (0.9%)
*Enterococcus faecalis*	0 (0.0%)	2 (0.5%)
*Enterococcus faecium*	1 (2.9%)	0 (0.0%)
*Escherichia coli*	28 (82.4%)	376 (87.6%)
*Klebsiella pneumoniae*	0 (0.0%)	21 (4.9%)
*Klebsiella oxytoca*	0 (0.0%)	2 (0.5%)
*Klebsiella ozaenae*	0 (0.0%)	1 (0.2%)
*Proteus mirabilis*	0 (0.0%)	1 (0.2%)
*Pseudomonas aeruginosa*	0 (0.0%)	2 (0.5%)
*Raoultella planticola*	0 (0.0%)	1 (0.2%)

Data are presented as number (percent).

### Prevalence of vesicoureteral reflux

Among all enrolled patients, confirmative imaging studies for VUR were performed on 259 patients (55.9%). The prevalence of VUR was significantly higher in the bacteremic UTI group compared to the non-bacteremic UTI group (59.3% vs. 30.6%; *P* = 0.003).

### Risk factor analysis for bacteremic UTI

**Figure 1:**
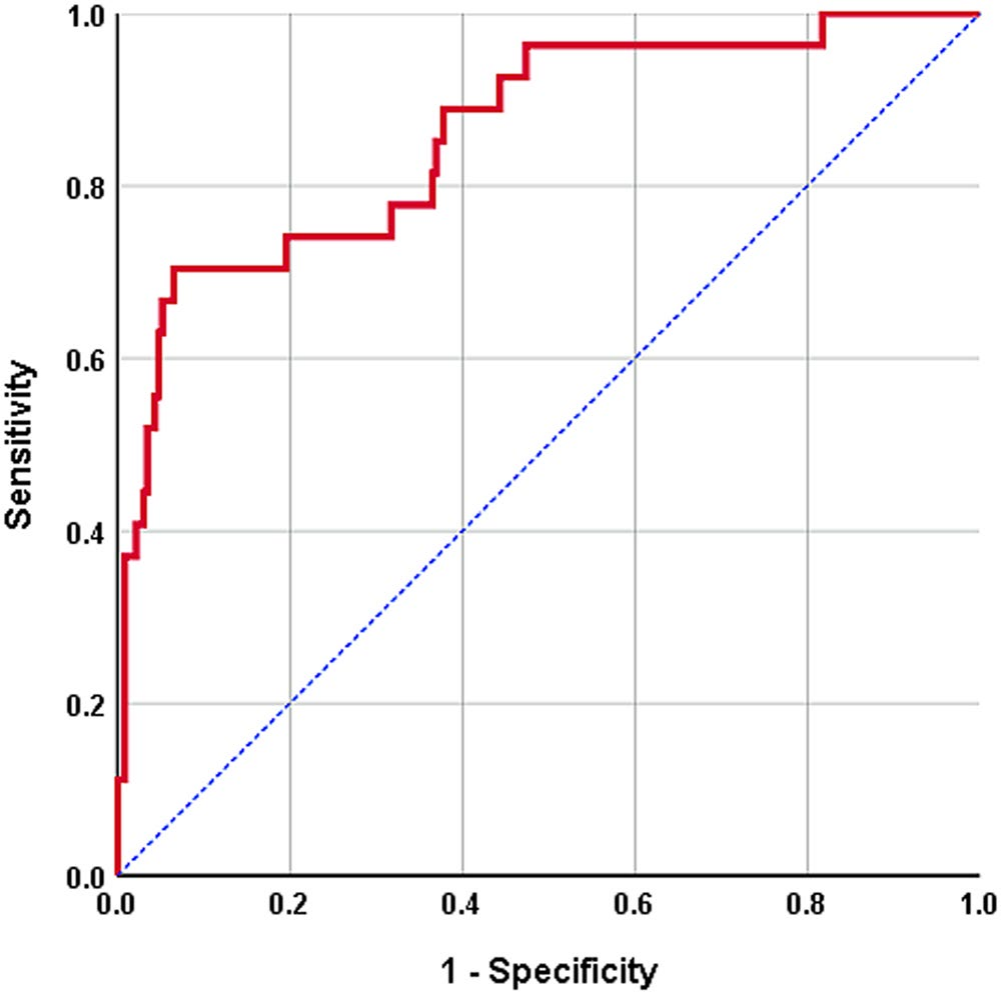
Receiver operating characteristic (ROC) curves for the multivariate model in discriminating bacteremic urinary tract infection from non-bacteremic urinary tract infection. The area under the ROC curve is 0.859.

Univariate logistic regression analyses showed that age ≤90 days; higher DNI, CRP, and creatinine levels; lower Hb and albumin levels; and the presence of VUR were significantly associated with increased risks of bacteremic UTI (Table 3). The multivariate model was based on 257 patients after removing missing values [n = 206; the missing values were from CRP (n = 3) and the confirmative imaging study of VUR (n = 204). One patient was had neither CRP nor imaging study of VUR]. On the basis of multivariate logistic regression analysis, age ≤90 days (odds ratio [OR]: 10.67, 95% CI: 2.90–39.20, *P* < 0.001); higher DNI (OR: 1.25, 95% CI: 1.08–1.44, *P* = 0.002), and CRP (OR: 1.02, 95% CI: 1.01–1.04, *P* < 0.001); and presence of VUR (OR: 3.66, 95% CI: 1.33–10.05, *P* = 0.012) were independent predictors of bacteremic UTI (Table 3). ROC analysis of our multivariate model revealed the diagnostic performance of bacteremic UTI was 0.859 (95% CI, 0.779–0.939; *P* < 0.001) (Fig. 1).

**Table 3:** Risk factors for bacteremic urinary tract infection in febrile infants with pyuria.

	*Univariate*			*Multivariate*		
Variables	OR	95% CI	*P*-value	OR	95% CI	*P*-value
Albumin (g/dL)	0.15	0.05–0.51	0.002	0.71	0.07–6.85	0.768
CRP (mg/L)	1.02	1.01–1.02	<0.001	1.02	1.01–1.04	<0.001
Cr (mg/dL)	13.57	1.31–141.13	0.029	19.38	0.68–552.59	0.083
DNI (%)	1.24	1.13–1.36	<0.001	1.25	1.08–1.44	0.002
Hb (g/dL)	0.66	0.46–0.94	0.022	1.67	0.88–3.15	0.116
Age						
≤ 90 days	4.12	1.92–8.83	<0.001	10.67	2.90–39.20	<0.001
> 90 days	reference					
VUR						
(+)	3.30	1.46–7.47	0.004	3.66	1.33–10.05	0.012
(–)	reference					

CI, confidence interval; CRP, C-reactive protein; Cr, creatinine; DNI, delta neutrophil index; Hb, hemoglobin; OR, odds ratio; VUR, vesicoureteral reflux. Odds ratio, 95% confidence intervals and *P*-values were calculated by binary logistic regression analysis.

### Cutoff values of laboratory tests for predicting bacteremic UTI

The ROC analysis revealed that optimal cutoff values for predicting bacteremic UTI of CRP and DNI alone were DNI alone >1.2% (AUC, 0.727; 95% CI, 0.684–0.768; *P* < 0.001; sensitivity, 61.8%; specificity, 77.9%; negative predictive value [NPV], 96.3%; positive predictive value [PPV], 18.1%) and CRP alone >45.1 mg/L (AUC, 0.680; 95% CI, 0.635–0.723; *P* < 0.001; sensitivity, 70.6%; specificity, 62.0%; NPV; 96.4% PPV, 12.9%), respectively (Supplementary Table S1 and Supplementary Fig. S1).

### Findings of DMSA renal scan

The results of DMSA scan during the acute stage (within seven days after admission) were obtained in 387(83.6%) patients [bacteremic UTI patients = 23 (67.6%, 23/34) vs. non-bacteremic UTI patients = 364 (84.8%, 364/429)]. Cortical defects were more frequently found in bacteremic UTI patients (60.9%) than non-bacteremic UTI patients (33.0%) during the acute stage (*P* = 0.033) (Supplementary Table S2).

However, the majority of enrolled patients were lost to follow-up; therefore, the results of the DMSA scan at six months after admission were only obtained for 23 patients [bacteremic UTI patients = 2 vs. non-bacteremic UTI patients = 21]. Most patients (91.3%, 21/23) showed resolution or improved status of cortical defects on follow-up DMSA scan (Supplementary Table S2). One patient who was initially diagnosed as a non-bacteremic UTI showed a renal scar on the follow-up DMSA scan.

### Proteinuria at follow up

The lack of long-term follow-up data for enrolled patients means that we could only obtain follow-up urinalysis at six-months [bacteremic UTI patients = 11 (32.4%, 11/34) vs. non-bacteremic UTI patients = 109 (25.4%, 109/429)] and one-year [bacteremic UTI patients = 14 (41.2%, 14/34) vs. non-bacteremic UTI patients = 33 (7.7%, 33/429)] after admission on follow-up visit (± 30 days). There was no significant difference in positive proteinuria cases in bacteremic vs. non-bacteremic UTI patients on the follow-up urinalysis (Supplementary Table S3).

### Renal function at follow up

eGFR was obtained from 53 patients (11.4%) [Bacteremic UTI = 9 (26.5%, 9/34) vs. Non-bacteremic UTI = 44 (10.3%, 44/429) until Jan 2020. The serum cystatin C concentration, eGFR, and number of cases with eGFR <90 mL/min/1.73 m^2^ showed no significant differences between bacteremic UTI and non-bacteremic UTI patients (Supplementary Table S4).

## Discussion

This study showed that age ≤90 days, higher levels of CRP and DNI, and the presence of VUR were significantly associated with concomitant bacteremic UTI in multivariate analysis. Our results also suggest that children with bacteremic UTI should be examined by VCUG or radionuclide cystourethrography because of the high prevalence (59.3%) of VUR.

Concomitant bacteremia or sepsis in UTI is associated with a longer length of hospital stay and higher morbidity and mortality rates in both adults or children^5,15^; thus, various studies have been conducted to identify early factors for predicting concomitant bacteremia.

One of the best known risk factors for concomitant bacteremic UTI is age. In pediatric studies, the rate of concomitant bacteremic UTI decreased with age^2–4,6,10^. Honkinen *et al*. reported that among patients <16 years of age with bacteremic UTI, 66% were aged 1 week to 3 months, 22% were aged 3–11 months, and 12% were aged ≥12 months^8^. Roman *et al*.^4^ reported an inverse relationship between the risk of bacteremic UTI and age, with a very low risk after 6 months. In accordance with previous studies, our results revealed higher numbers of infants aged ≤90 days in the bacteremic UTI group compared to in the non-bacteremic UTI group (70.6% vs. 36.8%). This tendency can be explained by the immature immune systems and more frequent hematogenous spread of infection in children of younger ages^12^.

In contrast, in adult patients, the rate of concomitant bacteremia with UTI is associated with old age^15,26,27^. Van Nieuwkoop *et al*. reported in a multicenter trial that bacteremia likely occurs 2.4 times in febrile patients with UTI > 65 years^27^. Elderly individuals are predisposed to sepsis because of their comorbidities, lengthy hospitalizations, reduced immunity, and functional limitations related to aging^28^.

Several studies have reported an association between sex and bacteremic UTI, males during the neonatal period^10^ and older ages^26^. However, beyond the neonatal period, many studies showed that sex did not increase the risk of bacteremic UTI^4,7,9,16^.

Patients with bacteremia had shorter fever duration prior to admission. Bacteremic UTI patients might present earlier than non-bacteremic UTI patients because of their poor general condition or non-bacteremic UTI patients might present to the hospital later in the disease’s course where the bacteremia have already disappeared. Unfortunately, we could not investigate what factors might influence bacteremia (e.g. the use of oral antibiotics prior to admission or hospital accessibility). Explaining this finding will require further future studies.

Well-known inflammatory markers, such as CRP and procalcitonin (PCT), have been studied to predict bacteremia because they can be rapidly evaluated compared to confirming positive blood cultures, which is time-consuming. Higher CRP levels have been frequently observed in pediatric patients with bacteremic UTI^7,8,16^, but there are no established optimal cutoff values for CRP. Kim *et al*.^16^ reported that an elevated CRP level (>0.5 mg/dL) independently increased the risk of bacteremic UTI (adjusted OR, 8.13). Velasco-Zuniga *et al*.^7^ studied 140 febrile infants aged 30–90 days with a positive urine culture and showed that CRP > 40 mg/L highly predicted adverse events (bacteremia or meningitis). These findings are similar to those of the present study, for which the optimal cutoff value of CRP alone was >45.1 mg/L for predicting bacteremic UTI.

PCT is a valid marker for diagnosing bacterial infections. Hernandez-Bou *et al*.^12^ studied afebrile infants aged 29–90 days with UTI and reported that the PCT level was significantly higher in infants with bacteremic UTI than in infants with non-bacteremic UTI (*P* = 0.031). The cutoff PCT of ≥0.7 ng/mL showed a sensitivity, specificity, PPV, and NPV of 87.5%, 71.1%, 8.3%, and 99.5%, respectively, as a predictor of bacteremia. In a prospective multicenter study involving 581 adults with UTI (median age, 66 years), van Nieuwkoop^27^ reported that a PCT cutoff value >0.25 μg/L was highly predictive of concomitant bacteremic UTI (sensitivity, 95%; specificity, 50%; PPV, 36%; NPV, 97%). The diagnostic accuracy of PCT for predicting bacteremia was 0.73 (95% CI: 0.68–0.77)^27^ to 0.82 (95% CI: 0.77–0.86)^12^ in the above studies. ROC analysis of our multivariate model revealed that the accuracy was 0.859 (95% CI, 0.779–0.939), which is higher than that of studies that only used PCT as a predictive factor. Unfortunately, we did not investigate the association between PCT levels and the presence of bacteremic UTI, and thus further studies are needed to confirm the predictive value of PCT in febrile infants for diagnosing concomitant bacteremic UTI at presentation.

DNI is a fraction of immature granulocytes previously reported as a useful marker for diagnosing bacteremia/sepsis with a higher overall diagnostic accuracy than that of traditional markers, such as PCT and CRP^29^. DNI can be determined by performing a complete blood count using an automated blood cell analyzer, and thus is rapid and inexpensive method for estimating bacteremia. In this study, DNI alone showed a moderate diagnostic capacity for detection of bacteremia (AUC, 0.727), with an optimal cutoff value of 1.2%. This is the first study to investigate the value of DNI for predicting bacteremia in infants with UTI. Ahn *et al*. reported that the best cutoff value of DNI for immunocompetent children (median age, 2.6 years) in predicting bacteremia, regardless of etiology, was 4.4%, with sensitivity and specificity values of 44.4% and 94.7%, respectively^30^. Additional studies are needed to determine age-appropriate, reliable cutoff values of DNI under various clinical conditions.

Albumin is also a potent prognostic marker of sepsis. A low albumin level has been also found in patients with concomitant bacteremic UTI^26,31^. In studies involving adults, Leibovici *et al*.^31^ identified a low serum albumin level as an independent predictor of bacteremia in hospitalized patients with UTI. Bahagon *et al*.^26^ also found that the serum albumin level was significantly low in patients with bacteremic UTI compared to in those with non-bacteremic UTI, but found no association in multivariate analysis. We observed that a lower albumin level increased the risk of bacteremic UTI in univariate analysis (unadjusted OR, 0.15), but not in the multivariate analysis.

A higher creatinine level was associated with bacteremic UTI in univariate regression analysis in our study, supporting previous findings^9,10^. However, a relationship in the multivariate model was not observed, similar to in a previous study^8^. In an adult study, Leibovici *et al*.^31^ identified high creatinine levels as an independent risk factor for bacteremic UTI in hospitalized patients. Megged^9^ retrospectively reviewed 464 culture-proven pediatric patients with UTI who were admitted to the emergency department (mean age, 52 ± 48 months) and found that among clinical variables (urine collection methods, age <3 months, sex, ethnicity, creatinine level, and urologic abnormality), creatinine (OR, 3.67; 95% CI, 1.69–8.11) was the only independent risk factor for bacteremia. The author explained that hypotension from bacteremia decreases blood flow of the kidney, resulting in increased creatinine levels^9^. The author included only WBC and creatinine as laboratory parameters; hence, the results would be different from our results.

An increased ALT level was suggested as an independent risk factor for bacteremic UTI. Kim *et al*.^16^ retrospectively reviewed 883 pediatric patients with acute pyelonephritis and reported that a high ALT level (>50 IU/L) increased the risk of concomitant bacteremia (OR, 2.22; 95% CI, 1.08–4.56; *P* = 0.03). The authors suggested a possible mechanism, such as active participation of hepatocytes in the immune response during sepsis. However, we found no differences in the ALT level between patients with bacteremic and non-bacteremic UTI (*P* = 0.756). This may be because of differences in the patient cohort and definition of the disease. The former study enrolled children with acute pyelonephritis, which was defined as satisfying both a positive urine culture and renal scintigraphy with DMSA. DMSA positivity indicates more severe inflammation caused by UTI; thus, children with a higher probability of more severe disease were enrolled in the study. Furthermore, the former study enrolled older patients than those in our study (36% were infants <3 months were; 43% were 4–12 months; 21% were >12 months).

Among the bacterial pathogens, *E. coli* is the most common pathogen in bacteremic UTI in both children and adults^4,10,15^. The relationship between the virulence genotype of *E. coli* strains and risk of bacteremic UTI was studied previously. Bonacorsi *et al*.^32^ compared *E. coli* isolates causing bacteremic and non-bacteremic UTI in 83 male infants younger than 90 days. The authors suggested that the absence of both hemolysin and antigen K1 are negative predictive factors for bacteremia.

An indwelling urinary catheter has been frequently identified as a risk factor for urosepsis in adult^26,33^. In a pediatric study, however, Roman *et al*.^4^ reported no significant difference between infants with bacteremic UTI and non-bacteremic UTI in terms of comorbidities, including immunodeficiency, neuromuscular disorders, and the presence of the indwelling urinary or central venous catheter. In our study, no enrolled patients had an indwelling catheter.

VUR is known to be predisposing for ascending kidney infection, renal scarring, and urosepsis in children^9,34^. A prevalence of 0.4–1.8% has been estimated for children without other concomitant conditions^35^. Swerkersson *et al*. reported VUR in 80 of 303 children (26%) younger than 2 years with a first-time, culture-verified UTI^36^. In the present study, VUR was detected nearly two-fold as frequently in patients with bacteremic UTI than in those with non-bacteremic UTI (59.3% vs. 30.6%; *P* = 0.003). Multivariate analysis revealed that if the infant had VUR, then the risk of bacteremic UTI was increased by approximately four-fold. Megged^9^ reported that children with bacteremic UTI (n = 26; mean age, 30 ± 49 months) were more inclined to have urogenital abnormalities than those with non-bacteremic UTI (n = 438; mean age, 53 ± 48 months) (13% vs. 5%; *P* = 0.04), but found no association in multivariate analysis.

Although the guidelines of the American Academy of Pediatrics do not recommend routine VCUG in every febrile infant with a first UTI, we suggest that imaging studies should be performed to evaluate the presence of VUR infants with bacteremic UTI.

A UTI may lead to acute renal parenchymal damage and permanent renal scarring^37^. Substantial scarring may lead to decreased kidney function and has been associated with subsequent hypertension, proteinuria, and end-stage renal disease^38–42^. However, the potential impact of concomitant bacteremia in UTI on both renal scarring and the development of renal insufficiency remains uncertain. In our study, cortical defects on DMSA scan were more frequently found in bacteremic UTI patients (60.9%) than non-bacteremic UTI patients (33.0%) during the acute stage, whereas most cases for whom follow-up DMSA was performed six months after UTI showed resolution or improved status of cortical defects (91.3%). Moreover, we found no difference in cases showing impaired renal function or proteinuria at follow-up. Previous studies regarding the long-term outcomes for children with UTI showed no significant decline in renal function after childhood UTI^39,43^. However, other long-term studies have shown that the onset of complications after UTI (e.g. hypertension, proteinuria, and chronic renal disease) may actually present after a few decades^38,40,42^. Additionally, we could not investigate the baseline eGFR and the follow-up data in our study were obtained in too few patients to generalize the results. Therefore, further large-scale studies are required to assess the long-term clinical outcome of bacteremic UTI patients, estimate their prognoses, and determine appropriate management.

The present study had several limitations. First, this was a retrospective study performed at a single, tertiary hospital. Therefore, the severity of disease may be skewed, and further multicenter studies are required to validate the study results. Second, data regarding previous antimicrobial use and hospitalizations were excluded because of the limited availability of this information. Third, the predictive capacity with other laboratory markers, such as PCT, could not be compared. Fourth, we could not assess the development of hypertension or the recurrence of UTI among enrolled patients.

The present study demonstrated the incidence and various laboratory risk factors for bacteremic UTI in infants aged <6 months at initial presentation. Further prospective studies are needed to confirm the diagnostic accuracy of these laboratory parameters under various patient cohorts and hospital settings to establish reliable cutoff values.

## Conclusions

The present study found that age ≤90 days, higher CRP and DNI levels, and the presence of VUR were significantly associated with bacteremia in infants with febrile UTI. These findings could be helpful to physicians in decision-making for the proper management of febrile infants with pyuria. Imaging studies such as VCUG are also recommended in infants with bacteremic UTI for the evaluation of VUR.

## Supplementary information

Supplementary information.

## Data Availability

The datasets used and/or analysed during the current study are available from the corresponding author on reasonable request.
