# Supplementary material for: Predictive factors for bacteremia in febrile infants with urinary tract infection
Source: Sci Rep. 2020 Mar 11;10:4469. doi: 10.1038/s41598-020-61421-4 (PMC7066144; doi:10.1038/s41598-020-61421-4)
Supplement: Supplementary file 1 — Supplementary information. [file 41598_2020_61421_MOESM1_ESM.docx]

**Supplementary Information**

**Predictive factors for bacteremia in febrile infants with urinary tract infection**

*Seo Hee Yoon^1^, HyunDo Shin^1^, Keum Hwa Lee^1,2,3^,* *Moon Kyu Kim^1^, Dong Soo Kim^1^, Jong Gyun Ahn^1^ and Jae Il Shin^1,2,3^*

^1^Department of Pediatrics, Yonsei University College of Medicine, Yonsei-ro 50, Seodaemun-gu, C.P.O. Box 8044, Seoul 120-752, Korea

^2^Division of Pediatric Nephrology, Severance Children’s Hospital, Seoul 03722, Korea

^3^Institute of Kidney Disease Research, Yonsei University College of Medicine, Seoul 03722, Korea

**Corresponding author:**Jong Gyun Ahn^‡^
Department of Pediatrics, Yonsei University College of Medicine

Yonsei-ro 50, Seodaemun-gu, C.P.O. Box 8044

Seoul 120-752, Korea

Tel: +82-2-2228-2057, Fax: +82-2-393-9118
Email: [JGAHN@yuhs.ac](mailto:JGAHN@yuhs.ac)

Jae Il Shin^‡^
Department of Pediatrics, Yonsei University College of Medicine

Yonsei-ro 50, Seodaemun-gu, C.P.O. Box 8044

Seoul 120-752, Korea

Division of Pediatric Nephrology, Severance Children’s Hospital

Seoul 03722, Korea

Institute of Kidney Disease Research, Yonsei University College of Medicine

Seoul 03722, Korea

Email: [SHINJI@yuhs.ac](mailto:SHINJI@yuhs.ac)

‡ These authors contributed equally to this work.

**Supplementary Table S1.** Cutoff levels for C-reactive protein (CRP) and delta neutrophil index (DNI) measurements, when evaluated as predictive markers for diagnosis of concomitant bacteremic urinary tract infection.

|  | **Cutoff value** | **Sensitivity %  (95% CI)** | **Specificity %  (95% CI)** | **PPV %** | **NPV %** | **+LR** | **-LR** |
| --- | --- | --- | --- | --- | --- | --- | --- |
| **CRP (mg/L)** | 45.1 | 70.6 (52.5–84.9) | 62.0 (57.2–66.6) | 12.9 | 96.4 | 1.86 | 0.47 |
| **DNI (%)** | 1.2 | 61.8 (43.6–77.8) | 77.9 (73.6–81.7) | 18.1 | 96.3 | 2.79 | 0.49 |

CI, confidence interval; CRP, C-reactive protein; DNI, delta neutrophil index; PPV, positive predictive value; NPV, negative predictive value; + LR, positive likelihood ratio; –LR, negative likelihood ratio.

**Supplementary Figure 1.** Receiver operating characteristic (ROC) curves for C-reactive protein (CRP) and delta neutrophil index (DNI) for discriminating bacteremic urinary tract infection from non-bacteremic urinary tract infection.

**Supplementary Table S2.** Comparison of renal cortical scintigraphy with technetium-99m (Tc-99m) dimercaptosuccinic acid (DMSA) findings between infants with bacteremia and non-bacteremia with febrile urinary tract infection.

|  | **DMSA findings** | **Bacteremic UTI** | **Non-bacteremic UTI** | ***P*-value** |
| --- | --- | --- | --- | --- |
| Acute stage (within seven days from admission) | Normal | 7 (30.4) | 219 (60.2) |  |
|  | Cortical defect | 14 (60.9) | 120 (33.0) | **0.033**^a^ |
|  | Renal scarring | 1 (4.3) | 18 (4.9) |  |
|  | Cortical defect + Renal scarring | 1 (4.3) | 7 (1.9) |  |
|  | Total | 23 | 364 |  |
| Follow up  at six months | Resolution | 0 (0.0) | 7 (33.3) |  |
|  | Improved | 2 (100.0) | 12 (57.1) |  |
|  | Unchanged | 0 (0.0) | 1 (4.8) | 0.704^a^ |
|  | Renal scarring | 0 (0.0) | 1 (4.8) |  |
|  | Total | 2 | 21 |  |

DMSA was performed within seven days of admission and follow-up DMSA was performed at six months after admission (±30 days). Data are presented as case numbers and percentages. Statistically significant differences were demonstrated using ^a^Chi-square tests. UTI, urinary tract infection.

**Supplementary Table S3.** Proteinuria at short term follow-up among infants with bacteremia and non-bacteremia with febrile urinary tract infection at admission.

| **Proteinuria** |  | **Bacteremic UTI** | **Non-bacteremic UTI** | ***P*-value** |
| --- | --- | --- | --- | --- |
| Six months after admission | + | 1 (9.1) | 2 (1.8) | .252^a^ |
|  | – | 10 (90.9) | 107 (98.2) |  |
|  | Total | 11 (100) | 109 (100) |  |
| One year after admission | + | 1 (7.1) | 1 (3.0) | .512^a^ |
|  | – | 13 (92.9) | 32 (97.0) |  |
|  | Total | 14 (100) | 33 (100) |  |

Follow-up urinalysis was performed at six months and one year after admission (± 30 days). Data are presented as case numbers and percentages. Statistically significant differences were demonstrated using ^a^Chi-square tests. UTI, urinary tract infection.

**Supplementary Table S4.** Follow-up renal function between infants with bacteremia and non-bacteremia with febrile urinary tract infection at admission.

| **Renal function** | **Bacteremic UTI  (n = 9)** | **Non-bacteremic UTI (n = 44)** | **Total (n = 53)** | ***P*-value** |
| --- | --- | --- | --- | --- |
| Follow-up time, months | 27.9 (32.4) | 30.7 (36.3) | 29.3 (35.1) | .972^a^ |
| eGFR (mL/min/1.73 m^2^) | 106.3 (84.5) | 127.8 (73.8) | 127.8 (71.0) | .476^a^ |
| Cystatin C (mg/L) | 1.06 (0.31) | 0.89 (0.21) | 0.89 (0.24) | .141^a^ |
| eGFR < 90 (mL/min/1.73 m^2^) | 2 (22.2) | 7 (15.9) | 9 (17.0) | .646^b^ |

Data are presented as median (interquartile range), case numbers, and percentages. Statistically significant differences were demonstrated using ^a^Mann–Whitney tests and ^b^Chi-square tests. eGFR, estimated glomerular filtration rate; UTI, urinary tract infection.
